# Editorial: Emerging talents in comparative immunology: 2022

**DOI:** 10.3389/fimmu.2023.1318852

**Published:** 2023-10-27

**Authors:** Yan-Jun Liu, Xin-Jiang Lu, Chris Hauton, Guan-Jun Yang, Jiong Chen

**Affiliations:** ^1^ State Key Laboratory for Managing Biotic and Chemical Threats to the Quality and Safety of Agro-products, Ningbo University, Ningbo, Zhejiang, China; ^2^ Department of Physiology, Zhejiang University School of Medicine, Hangzhou, Zhejiang, China; ^3^ Department of Hepatobiliary and Pancreatic Surgery of the First Affiliated Hospital, Zhejiang University School of Medicine, Hangzhou, Zhejiang, China; ^4^ School of Ocean and Earth Science, National Oceanography Centre, University of Southampton, Southampton, United Kingdom; ^5^ Laboratory of Biochemistry and Molecular Biology, School of Marine Sciences, Ningbo University, Ningbo, China; ^6^ Key Laboratory of Aquacultral Biotechnology Ministry of Education, Ningbo University, Ningbo, China

**Keywords:** comparative immunology, LECT2, P2X receptor, germinal center, MCSF-2

Comparative immunology (CI) is discipline that focuses on the immune systems of different, often non-model, organisms, aiming to understand the similarities and differences in how they defend against pathogens and maintain immune homeostasis (1, 2). Comparative studies allow researchers to examine how immune systems have evolved across different species over time via identifying conserved immune components and pathways, from which scientists can infer ancestral immune mechanisms and track the emergence of novel adaptations (3). Secondly, CI helps to identify key immune processes that are shared among species via studying diverse organisms with different immune systems, such as invertebrates. Researchers can elucidate fundamental immune mechanisms that might be conserved across more complex organisms, including humans (4). Thirdly, CI also aids in unraveling variations in disease susceptibility among species. Researchers gain insights into factors that contribute to resistance or susceptibility to infectious diseases via comparing immune responses to pathogens and studying host-pathogen interactions (5). Fourthly, CI contributes to the development of vaccines (6). By examining immune responses in different species, researchers can identify effective vaccine strategies based on their shared immune competences, and optimize immunization protocols. Finally, comparative studies have biomedical relevance as well, helping in the identification of potential targets for therapeutic intervention (7). Understanding immune pathways in non-human models can shed light on human immune disorders and provide insights into novel treatment approaches.

This Research Topic highlights the significant contributions of emerging comparative immunologists in deepening our knowledge of immune system biology, evolution, and disease-related processes across various species. The acceptance of 5 articles from 28 authors around the world demonstrates the growing interest in this field:

Matz and Dooley explored the evolutionary foundations of germinal centers (GCs) in endothermic vertebrates such as mammals and select avian species (8), where they serve as specialized sites for B cell selection and affinity maturation, resulting in the generation of memory B cells and plasma cells (9). However, GCs have not been observed in ectothermic jawed vertebrates like fish, reptiles, and amphibians. Nevertheless, research spanning several decades suggests that these vertebrates can generate antigen-specific B cell responses with varying levels of affinity maturation. Matz and Dooley critically collected and evaluates the available data, supporting the notion that the fundamental mechanisms governing B cell selection may exhibit a higher level of evolutionary conservation throughout vertebrate phylogeny than initially presumed. Furthermore, investigations conducted in established mammalian model systems, as well as comparative models, give rise to an intriguing query regarding the selective advantages conferred by GCs to endothermic vertebrates. This inquiry stems from the apparent superfluity of GCs in generating the essential functional constituents of humoral adaptive immune responses in jawed vertebrates of ectothermic nature. By delving into this question, their review contributes to our comprehension of the evolutionary significance of GCs in immune system development across different vertebrate taxa.

Asthma is a prevalent condition globally, significantly impacting the quality of life and leading to frequent hospitalizations (10). Aldakheel et al.‘s study shed light on the potential coagulation abnormalities in individuals with asthma through investigating the association between immune cell ratios and coagulation markers. The study’s findings suggest that monitoring the ENR and d-dimer levels can be helpful in predicting disease prognosis and the development of coagulation abnormalities in asthma patients. This research offers valuable insights into the correlation between immune cell ratios and coagulation markers in asthma, thereby enhancing our comprehension of disease progression and related complications. These insights may lead to improved management strategies and better outcomes for individuals with asthma, ultimately improving their quality of life and reducing hospitalizations. It is hoped that further research will be conducted in this area to develop more effective treatments for asthma patients.

Leukocyte cell-derived chemotaxin-2 (LECT2) is a secreted factor with multiple functions in various physiological and pathological processes (11–13). Zhu et al. focused on elucidating the emerging its functions in immune-related diseases, aiming to unravel the underlying mechanisms and explore its therapeutic potential. LECT2 is a versatile protein that exhibits high sequence similarity among distinct vertebrates, making it an ideal subject for comparative studies to explore its functions in immune processes and related diseases. It binds to various cell surface receptors, including CD209a, Tie1, and Met, in different cell types, but due to functional and signaling heterogeneity, its specific involvement in diverse immune pathogenic conditions in various tissues is still not fully understood. Misfolded LECT2 is associated with amyloidosis, a condition characterized by the buildup of insoluble fibrils in vital organs like the kidney, liver, and lung. This comprehensive review provides insights into the structural features, omnifarious functions, and mediated signalings of LECT2 in immune diseases, along with potential therapeutic applications in preclinical and clinical trials. By presenting aI comprehensive perspective on the association between LECT2 and immune diseases, this review aims to facilitate the discovery of drugs or probes targeting LECT2 for improved diagnosis and treatment of immune-related diseases.

P2X receptors are a family of ligand-gated ion channels that are activated by extracellular ATP. In mammals, these receptors play crucial roles in inflammation and immune response (14, 15). However, their functions in fish species have not been well understood. Sun et al. conducted a study to characterize P2X receptor homologues in spotted sea bass (*Lateolabrax maculatus*) and investigated their tissue distributions, expression patterns, and functional roles in regulating inflammation-associated genes. The researchers identified and characterized four P2X receptor homologues in the spotted sea bass, namely LmP2X -2, -4, -5, and -7. They used real-time quantitative PCR (qPCR) and luciferase assay to examine the expression levels of these receptors. The topological structures, genomic organization, and gene synteny of these receptors were found to be similar to those observed in other previously studied species. The four receptor genes were constitutively expressed in tested tissues, and their expression was significantly induced upon stimulation with *Edwardsiella tarda* and/or pathogen-associated molecular patterns (PAMPs) *in vivo*. In primary head kidney leukocytes of *L. maculatus*, LmP2X2 and LmP2X5 showed induction upon stimulation with PAMPs and/or ATP. In addition, when overexpressed in HEK 293T cells, LmP2X2, LmP2X4, and LmP2X7 exhibited differential upregulation of pro-inflammatory cytokines and apoptosis-related genes upon ATP stimulation. Furthermore, the activation of any one of the four receptors led to an upregulation of *NF-κB* promoter activity, indicating their involvement in modulating the expressions of pro-inflammatory cytokines through the NF-*κ*B pathway. This study provides novel evidence for the presence and function of P2X receptors in the inflammatory immunity of fish species, specifically in the spotted sea bass.

MCSF-2 is a vital cytokine that regulates the differentiation, proliferation and survival of cells in the monocyte/macrophage lineage in vertebrates (16). In fish, two unique isoforms of MCSF have been discovered, each with specific gene organization and expression patterns. However, there is limited information available on their immunomodulatory and functional properties. Gouife et al. conducted a study on goldfish MCSF-2 (gf*MCSF-2*), examining its molecular characteristics, gene expression patterns, and functional properties. They found significant differences between gfMCSF-1 and gfMCSF-2, suggesting that they are distinct genes rather than isoforms. Sequence alignment revealed similarity between gfMCSF-2 and MCSF-2 homologs from other fish species. Expression analysis showed varying levels of gfMCSF-2 expression in different goldfish tissues, with the spleen showing the highest expression. In goldfish head kidney leukocytes (HKLs), gfMCSF-2 expression was influenced by chemical stimuli and bacterial infection, being upregulated by lipopolysaccharide (LPS) and live *Aeromonas hydrophila*. The researchers successfully produced recombinant gfMCSF-2 (rgMCSF-2), which promoted cell proliferation in HKLs. Flow cytometric analysis demonstrated rgMCSF-2-induced differentiation of sorted leukocytes. Additionally, rgMCSF-2 treatment upregulated *TNFα* and *IL-1*β mRNA levels and affected the expression of transcription factors such as *MafB*, *GATA2*, and *cMyb* in a time-dependent manner. This study advances our comprehension of gfMCSF-2’s immune regulatory mechanisms and provides foundational knowledge for further research on its role in fish immune responses.

## Conclusion

In sum, this Research Topic collected 5-emerging talents’ works in comparative immunology in 2022. These emerging talents in comparative immunology have significantly advanced our understanding of immune system evolution, host-microbiome interactions, and innate immunity. Their research has paved the way for further investigations in these areas and holds implications for human health, evolutionary insights, and biomedical applications.

## Author contributions

Y-JL: Writing – original draft, Writing – review & editing. X-JL: Conceptualization, Investigation, Resources, Writing – review & editing. CH: Conceptualization, Writing – review & editing, Visualization. G-JY: Conceptualization, Writing – review & editing, Funding acquisition, Project administration, Supervision, Writing – original draft. JC: Funding acquisition, Supervision, Writing – review & editing.
